# CRABP1 Signalosomes in Non-Canonical Actions of Retinoic Acid—Maintaining Health and Preventing Thyroid Dysfunction in Aging

**DOI:** 10.3390/endocrines6020026

**Published:** 2025-06-03

**Authors:** Jennifer Nhieu, Fatimah Najjar, Li-Na Wei

**Affiliations:** Department of Pharmacology, University of Minnesota Medical School, Minneapolis, MN 55455, USA

**Keywords:** CRABP1, retinoic acid, signalosome, disease, thyroid, stress, aging

## Abstract

Retinoic acid (RA) exerts biological effects through RA receptors (RARs) to regulate transcription. RA also elicits rapid, RAR-independent (noncanonical) activities mediated by Cellular RA Binding Protein 1 (CRABP1) to modulate cytosolic signaling. CRABP1 functions by forming protein complexes, named CRABP1 signalosomes, to modulate signal propagation in a cell type-specific manner. This review summarizes multiple CRABP1 signalosomes and their physiological functions. CRABP1 knockout (CKO) mice develop multiple phenotypes progressively throughout the lifespan. These include altered brain function, obesity, and insulin resistance starting at young adult stages, increased vulnerability to heart failure and altered serum exosome profiles in midlife, and motor deterioration and thyroid dysfunction (hypothyroidism) in later life. The mouse *Crabp1* gene is tightly regulated by multiple epigenetic mechanisms, whereas human *CRABP1* gene dysregulation is associated with multiple human diseases in which age is an important factor. Further, CRABP1 expression in human and mouse thyroid glands gradually increases with aging. This underscores the clinical relevance of CRABP1 signalosomes in maintaining health and the functions of certain cells/organ systems, especially in the thyroid and during the aging process. The CRABP1 sequence is highly conserved, likely due to its functional constraint in forming various signalosomes; its tight regulation ensures proper expression of CRABP1 required for the forming of various signalosomes critical to the health and functions of multiple cell types/organ systems. Finally, CRABP1-specific (without activating RARs) signaling pathway-selective compounds have been designed. It may be an attractive therapeutic strategy to exploit these CRABP1-specific compounds to modulate selective signaling pathways in certain disease conditions, such as thyroid dysfunction, to maximize efficacy while minimizing retinoid toxicity.

## 1. Introduction

Retinoic acid (RA) constitutes a group of active metabolites of Vitamin A (retinol), regulating a wide range of biological processes such as cell differentiation, proliferation, developmental processes, and the functions of major organ systems [1]. These activities are largely attributed to canonical RA signaling, where various forms of RA bind to nuclear RA receptors (RARs) or Rexinoid receptors (RXRs) to regulate gene transcription. Typically, RAR/RXR-mediated activities of RA irreversibly change the fate/function of the cells by regulating their genomes, a process that takes a long period of time to complete (hours or days) [2]. However, non-genomic RA activity mediated by RARs localized to neuron dendrites [3] or through the direct binding of RA to protein kinase C [4] has been reported.

Recent studies have established that RA, specifically the predominating form, *all-trans* RA, exerts “non-canonical effects”, defined as follows: (a) RAR/RXR-independent, (b) occurring on a rapid timescale (within minutes), and (c) localized to the cytosol [5,6]. This RA non-canonical activity functions to regulate specific cytosolic signaling pathways in particular cell types in a generally dynamic and reversible manner. These mostly cell type-specific, non-canonical activities of RA are mediated, primarily, by a highly conserved cytosolic protein named Cellular Retinoic Acid Binding Protein 1 (CRABP1) [6,7]. CRABP1 has been found to be important for maintaining the function and health of various organs/tissues/cells, including adipose tissues [8,9], the brain [10], the heart [11,12], spinal motor neurons (MNs) [13,14], and the thyroid gland [15]. Experimental and clinical data have shown that CRABP1 mainly plays a protective role in guarding certain crucial organ systems where cellular health and functional maintenance are critical, especially at certain stages of adulthood and during the aging process. Deleting the *Crabp1* gene in mice (CKO mice) caused age-dependent deleterious consequences throughout their lifespan. The CKO phenotypes progress from young to old ages, starting with enhanced memory function from young adult stages [10]; increased vulnerability to obesity/insulin resistance [8], heart failure [11], and altered serum exosome profiles [16,17] in midlife; and severely deteriorated motor activity [14] and thyroid function that causes primary hypothyroidism [15] in late adulthood.

Molecular and cell biology experiments have determined that within an organ where the *Crabp1* gene is active, CRABP1 is expressed only in certain cell types. In these CRABP1-positive cells, CRABP1 forms protein complexes with, and modulates the propagation of, specific signaling pathways and their components, thereby maintaining the health and function of these cells [7]. As of today, our studies have identified multiple CRABP1-containing complexes that can modulate specific signaling pathways, referred to as “CRABP1 signalosomes” [6,7]. In the following sections, these various CRABP1 signalosomes and their functions are described. Additionally, the implications of CRABP1 signalosomes in maintaining cell/organ functions and its clinical relevance to human health, particularly in aging and with an emphasis on thyroid health and dysfunction, are also discussed.

## 2. CRABP1 Signalosomes

**Figure 1.**
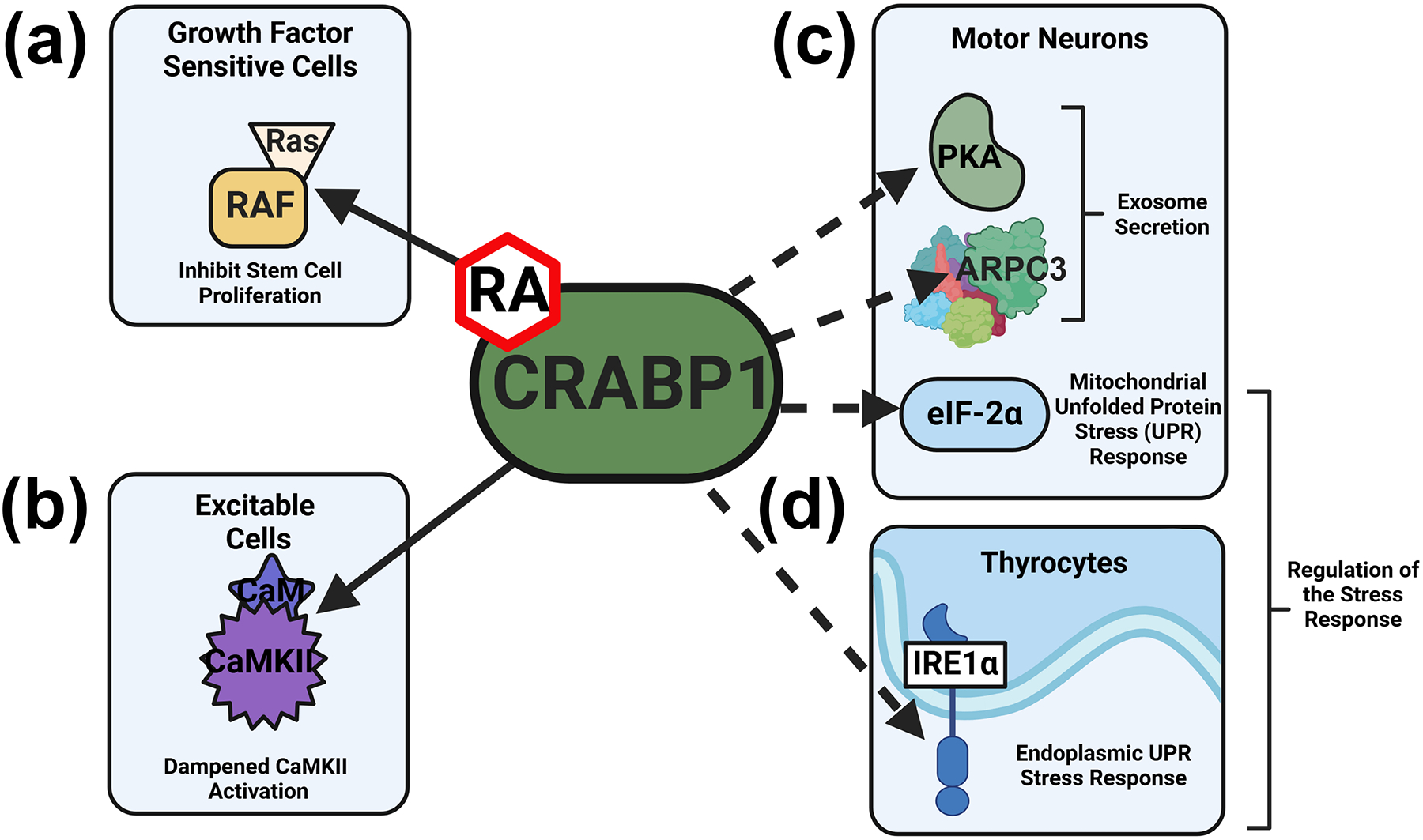
Established and putative CRABP1 signalosomes. (**a**,**b**) CRABP1-RAF and CRABP1-CaMKII signalosomes and their physiological functions in growth factor sensitive (**a**) and excitable cells (**b**). (**c**) In MNs, CRABP1-PKA and CRABP1-Arp2/3 signalosomes can function in modulating exosome secretion, and the CRABP1-eIF2α signalosome may act to modulate mitochondrial UPR. (**d**) In thyrocytes, CRABP1-IRE1α signalosome can act to modulate UPR to resolve ER stress. Solid lines depict two characterized CRABP1 signalosomes and dashed lines indicate CRABP1 signalosomes that remain to be further characterized. In vulnerable cells, CRABP1-eIF2α and CRABP1-IRE1α signalosomes can function to modulate stress responses.

CRABP1 is a highly conserved, RA-binding cytosolic protein that binds RA with a high affinity (K_d_ < 1 nM) [18–21]. Recent studies of the *Crabp1* gene knockout (CKO) mouse model and biochemical studies of CRABP1-containing protein complexes have shed light on the functional roles of CRABP1 and its mechanisms of action. As mentioned above, CKO mice progressively develop a spectrum of phenotypes along their lifespan [8,10,11,14–17]. These phenotypes were attributed to the ability of CRABP1 to form signalosomes with a component(s) of a cell-specific signaling pathway to modulate signal propagation [22,23]. Biochemical studies identified multiple types of CRABP1 signalosomes with functions in different cell types (Figure 1). Biophysical studies have revealed novel interaction surfaces on the protein structure of CRABP1, which facilitate direct binding with different interaction partners to modulate these cell-specific signaling pathways [22,23]. Based on these experimental data and bioinformatic analyses of human data, we propose that CRABP1 acts as a versatile scaffold protein, enhanced by RA, through the formation of “CRABP1 signalosomes” to modulate various signaling pathways that ultimately maintain the function and/or health of cells in specific organs/tissues.

A possible reason for the high conservation of CRABP1’s primary amino acid sequence across species (>99%) [7] is the evolutionary pressure to preserve these interaction surfaces, which are essential for the binding of partners to form specific CRABP1 signalosomes. In fact, throughout evolution, CRABP1 has maintained the same amino acid sequence in all the higher vertebrate species examined, allowing only one substitution occurring at position 86, which can be an Ala or a Pro [7]. Therefore, the sequence of CRABP1 is likely conserved because of its functional constraints. Currently, experimental data have revealed at least six types of CRABP1 signalosomes, these are described in the following sections.

### 2.1. CRABP1-RAF Signalosome (MAPK Signaling) in Dampening Stem Cell Proliferation

CRABP1 is highly expressed in all the stem cell types that have been examined, including numerous tumors, established embryonal carcinoma (EC) cells [24], embryonic stem (ES) cells [5], and neural stem cells (NSC) [10]. Studies have concluded that, in stem cells, CRABP1 acts to negatively modulate their proliferation by providing a “break” to dampen Mitogen Activated Protein Kinase (MAPK) signal propagation [5,10]. In the presence of RA, the growth signal-dampening effect of CRABP1 is rapidly enhanced (non-canonical activities) to first slow down proliferation, preparing cells for the canonical activities of RA that induce cell differentiation. This rapid growth-dampening effect of CRABP1/RA relies on its direct interaction with Rapid Accelerated Fibrosarcoma (RAF) kinase to form the CRABP1-RAF signalosome. RAF is a cell membrane-anchored kinase activated by the mitogenic signal Ras GTPase [25]. When CRABP1 interacts with RAF, it also competes out the Ras GTPase; thus, mitogenic signals can no longer effectively activate the MAPK signaling cascade, and cell growth is inhibited. Therefore, the CRABP1-RAF signalosome negatively modulates growth factor stimulated cell proliferation, ensuring the homeostasis of stem cell proliferation and differentiation to maintain a healthy stem cell pool. Interestingly, the CKO mice have improved memory functions associated with an enlarged NSC pool and increased neurogenesis in the hippocampus [10]. Further, clinical studies have reported CRABP1 as a tumor suppressor [26–34].

### 2.2. CRABP1-CaMKII Signalosome in Dampening Overexcitation-Induced Cellular Toxicity

In excitable cells like neurons and cardiomyocytes, CRABP1 directly interacts with calcium (Ca^2+^)/calmodulin-dependent kinase 2 (CaMKII), an enzyme critical to Ca^2+^ signaling/handling that is highly enriched in both cardiomyocytes [35] and neurons [36,37]. CaMKII regulates cardiomyocyte contraction and long-term potentiation in neurons. Both of these processes are dependent on proper Ca^2+^ signaling, which is critical in general to the function and health of these excitable cells [38]. CaMKII activation occurs following Ca^2+^ influx because Ca^2+^ binds calmodulin (CaM) to activate CaMKII. It appears that CRABP1 also competes with CaM for CaMKII binding, thereby dampening Ca^2+^/CaM-activated CaMKII activity. In these excitable cells, over-activated CaMKII is a major trigger of cell death/damage [39,40]; therefore, by dampening CaMKII over-activation, CRABP1 plays a protective role in maintaining the health of both cardiomyocytes and neurons, especially spinal MNs, which are constantly stimulated for routine motor function. Supporting the notion that CRABP1 is a protective player in excitable cells, CKO mice develop, spontaneously, cardiomyopathy [11] and then motor deterioration [14] at mid-adult stages. Further, human studies have reported that in several subtypes of ALS, *CRABP1* is the most downregulated gene [41,42], which may contribute to the increased vulnerability to MN degeneration observed in ALS patients. Our animal studies identified a role for the CRABP1-CaMKII signalosome in spinal MNs. Investigating the relevance to other neuronal types where both CaMKII and CRABP1 are highly expressed would be of great interest. For example, CaMKII is recognized for its role in long-term potentiation, a process also critical to learning and memory [43]. It is important to determine whether CRABP1-CaMKII signalosomes function in this context, which would provide important insights into the precise roles of the CRABP1-CaMKII signalosome in the central nervous system.

### 2.3. CRABP1-PKA and CRABP1-Arp2/3 Signalosomes in Modulating Exosome Secretion

Studies of CRABP1-silenced cells showed that their secreted exosome numbers were significantly reduced as compared to control cells [17]. The results suggested a functional role for CRABP1 in positively modulating exosome secretion. Further biochemical studies using immunoprecipitation mass spectrometry (IP-MS) and subsequent validation through in vitro interaction studies allowed the identification of two CRABP1-interacting proteins, protein kinase A (PKA) and the actin-related protein 2/3 (Arp2/3) complex involved in exosome secretion [44,45]. While our preliminary data have shown the functional outcome of deleting CRABP1 in terms of exosome secretion and the formation of CRABP1-PKA and CRABP1-Arp2/3 complexes, the exact mode of these CRABP1 signalosomes in exosome secretion remains to be rigorously determined. Importantly, CKO mice indeed have a very different blood exosome profile as compared to wild-type mice, including a reduction in the number of circulating exosomes [17] and an expansion of an exosome subpopulation carrying the pro-inflammatory protein RIP140 [16]. The exact steps in exosome secretion, such as vesicle formation, sorting, trafficking, and secretion, that can be modulated by CRABP1 remains to be determined. Interestingly, exosome secretion has also been implicated in various human diseases, ranging from cancer to neurodegeneration to metabolic and immune disorders, among others [46]. Thus, the specific contribution of CRABP1-PKA and/or CRABP1-Arp2/3 signalosomes in exosomes secretion in the context of disease vulnerability is of great interest for future studies.

### 2.4. CRABP1-eIF2_α_ and CRABP1 IRE1_α_ Signalosomes in the Cellular Stress Response

In response to stress, eukaryotic translation initiation factor 2 alpha (eIF2α) activation by phosphorylation is important for halting global protein synthesis and executing the selective translation of the proteins important for adaption and recovery [47]. This process is referred to as the Integrated Stress Response (ISR) and converges on the phosphorylation of eIF2α (p-eIF2α) mediated by upstream eIF2 kinases: protein kinase RNA-like endoplasmic reticulum kinase (PERK), general control nonderepressible 2 kinase (GCN2), heme-regulated eIF2α kinase (HRI), and protein kinase R (PRK) to manage stress [48]. A CRABP1-eIF2α complex has been detected in in vitro and mass spectrometry experiments, suggesting the formation of the CRABP1-eIF2α signalosome (unpublished). Interestingly, eIF2α hypo-phosphorylation (inactivation) was detected in spinal cord tissues of CKO mice, consistent with their defect or failure in the execution of mitochondrial-unfolded protein response (mt-UPR), a process associated with ISR [49,50]. Overall, these results suggest that the CRABP1-eIF2α signalosome may be involved in the modulation of stress response pathways, especially in neurons that are particularly vulnerable to stress, such as MNs [51]. Importantly, CKO MNs indeed have elevated stress burdens [50] and progressively deteriorated neuromuscular junctions as animals age [14]. Therefore, it is reasonable to speculate that the CRABP1-eIF2α signalosome may enhance the cell’s stress response capacity, thereby providing a protective mechanism when cells are under stress. Optimizing the stress response in particularly vulnerable cell types such as MNs is critical to their adaptation to physiological and/or environmental stressors, which is crucial for their health and function.

Inositol-requiring enzyme 1α (IRE1α) is a highly conserved sensor of accumulated unfolded proteins in the endoplasmic reticulum (ER) and a major executor of the ER-UPR. Initially, IRE1α is auto-phosphorylated through the binding of unfolded proteins, thereby activating transcription factors to degrade these unfolded proteins and increase protein secretion and autophagy [52]. Under chronic ER stress, persistent IRE1α activity, marked by hyper-phosphorylation, leads to the activation of cell death programming. Hyper-phosphorylation of IRE1α shifts the stress response from pro-survival signaling to a pro-apoptotic pathway through factors such as Caspase-2 activation [52]. The detection of a CRABP1-IRE1α complex in thyrocytes (unpublished) would indicate the presence of a CRABP1-IRE1α signalosome that may modulate the ER stress response by activating the ER-UPR pathway to resolve stress and promote cell survival [53,54]. Interestingly, CKO thyrocytes of old animals (older than 9 months) exhibit IRE1α hyper-phosphorylation; further, CKO thyrocytes are severely damaged (unpublished) and their thyroid hormone secretion is reduced [15]. Presumably, in thyrocytes, lacking CRABP1 might promote apoptosis, in part, through increasing hyper-phosphorylation of IRE1α. This may result from the loss of the CRABP1 signalosome-modulated IRE1α phosphorylation. With these observations, we suspect that the CRABP1-IRE1α signalosome may play a role in protecting certain cells, such as thyrocytes, from unwanted apoptosis (discussed further in Section 4).

The stress response is also highly implicated in numerous human diseases. For instance, decreases in p-eIF2α, due to loss-of-function mutations in its upstream kinases and regulators, result in neurodevelopmental defects, neurodegeneration, glucose intolerance, or skeletal defects [55]. For IRE1α, its over-activation has been implicated in autoimmune diseases [56], neurodegeneration [57], and obesity [58]. Hyper-activated or prolonged IRE1α activity may shift the system towards destructive, pro-apoptotic pathways. Interestingly, reduced CRABP1 expression and elevated IRE1α activation have been reported in autoimmune disorders such as vitiligo [7,56] and inflammatory bowel disease [7,56], as well as in the neurodegenerative disorder ALS [7,57]. These findings suggest that insufficient *CRABP1* expression may result in a failure to properly regulate IRE1α activity in stress-sensitive or disease-prone cells/tissues. In most cells, coordinated stress responses provide an important protective mechanism. The inability of certain cells to properly engage either eIF2α (CRABP1-eIF2α signalosome) or IRE1α (CRABP1-IRE1α signalosome) when *CRABP1* is reduced or absent would disrupt this important protective mechanism in certain cell types/organs that are particularly sensitive to various stressors and may contribute to disease. While CRABP1-eIF2α and CRABP1-IRE1α complexes can be detected in certain cell types, how CRABP1-eIF2α and CRABP1-IRE1α signalosomes act to modulate stress responses and/or apoptotic cell death remains to be determined.

Our previous studies have shown that RA enhances CRABP1’s interactions with RAF [23] and CaMKII [12] to dampen their enzyme activity, thereby preventing their over-activation. Therefore, it is reasonable to speculate that RA may also enhance CRABP1’s ability to inhibit IRE1α activity. We propose that RA, in general, may bolster CRABP1’s ability to recruit interaction partners, thereby enhancing its function in modulating cell signaling. However, the precise role of RA in regulating the CRABP1–IRE1α signalosome remains to be rigorously determined.

## 3. Regulation of the CRABP1 Gene

The data reviewed above have established important functional roles for CRABP1 in various cell types/organs at certain critical stages of an animal’s lifespan. For this, the expression of the *Crabp1* gene would require a rigorously guarded regulatory mechanism in specific cell types and/or at certain states/stages of cellular maturation or organ functions. Interestingly, extensive molecular studies of cell lines and animal models have consistently shown tightly regulated epigenetic mechanisms guarding the expression of the mouse *Crabp1* gene [59]. More recently, bioinformatic analyses revealed gradual changes in the expression of the human *CRABP1* gene along the human lifespan, particularly in certain organs/tissues, as well as significantly altered CRABP1 expression levels in multiple diseased conditions.

### 3.1. Epigenetic Regulation of the Mouse Crabp1 Gene

Molecular studies of the cloned mouse *Crabp1* gene first revealed a very interesting feature of its promoter, that it is a Sp1-containing “housekeeping” gene with several conserved regulatory elements in its upstream region responsible for its up- and downregulation [24,60]. These include a pair of “direct repeat 4s (DR4s)” which are a “thyroid hormone response element” (TRE) responsible for its thyroid hormone/RA induced upregulation, a highly G/C-rich region which spans the basal promoter and is sensitive to DNA methylation for its epigenetic silencing, and the ability of this promoter to form a chromatinloop mediated by the Mediator 1 chromatin-remodeling complex that is required for its induction by RA and thyroid hormones [24,60–64]. Based on in vitro molecular studies of this gene and validation using in vivo LacZ reporter cell lines and transgenic mouse models [60], it appears that the *Crabp1* gene is expressed at a very low basal level in most cell types examined, but that it can be rapidly upregulated by several factors, including RA, thyroid hormones, and alcohol [60,63,65]. This gene is also sensitive to DNA methylation-mediated gene silencing that triggers the formation of a nucleosome array on its basal promoter [64]. Thus, the *Crabp1* gene, while weakly expressed in many cell types through its house-keeping promoter, can be bidirectionally regulated, i.e., it can be silenced by DNA methylation/nucleosome formation (a heterochromatin state) and activated by thyroid hormones that trigger chromatin looping to resolve nucleosomes (an euchromatin state).

To demonstrate the dynamic, bidirectional regulation of the *Crabp1* gene, we have exploited a mouse adipocyte differentiation cell culture model where the *Crabp1* gene can undergo a series of gene activation and silencing events, progressing along the adipocyte differentiation process [61,64]. This in vitro model has demonstrated its basal expression state (for its housekeeping function) in a preadipocyte stage, which corresponds to the stage of stem/progenitor cells where CRABP1 acts to modulate growth signal inputs for proper proliferation. Upon differentiation to adipocytes, which require thyroid hormones, this gene is activated. However, upon the further expansion of adipocytes when fat begins to accumulate, the *Crabp*1 gene is gradually silenced through DNA methylation, chromatin remodeling, and nucleosome formation [64]. However, it remains unclear whether CRABP1 has a direct causal relationship with fat accumulation/adipocyte expansion in vivo. Importantly, data from a *Crabp1*-lacZ transgenic mouse model have provided in vivo evidence for “epigenetic regulation” of the *Crabp1* gene by several triggers, including alcohol, RA, and thyroid hormones [62,65].

This series of studies using molecular and reporter approaches have concluded that the expression of the *Crabp1* gene can be affected by multiple physiological/pathological inputs. Under normal physiological conditions, this gene engages various regulatory events to guard its temporally and spatially specific, and highly regulated, expression pattern. This is consistent with its versatile functional roles in forming cell-specific signalosomes to modulate distinct signaling pathways critical to the health/physiological functions of multiple cell types/organs. CRABP1 signalosomes play protective roles in these cells/organs and are important for animals’ post-natal health and functional maintenance along their lifespan. The mouse *Crabp1* gene can be regulated by various physiological (such as endocrine)/pathological (such as alcohol and obesity) factors; but it remains unknown whether and how it may be regulated by stress signals. This is also an important area to be further investigated.

### 3.2. Age-Dependent Changes in the Expression of CRABP1 in Human and Mouse Thyroid Glands

**Figure 2.**
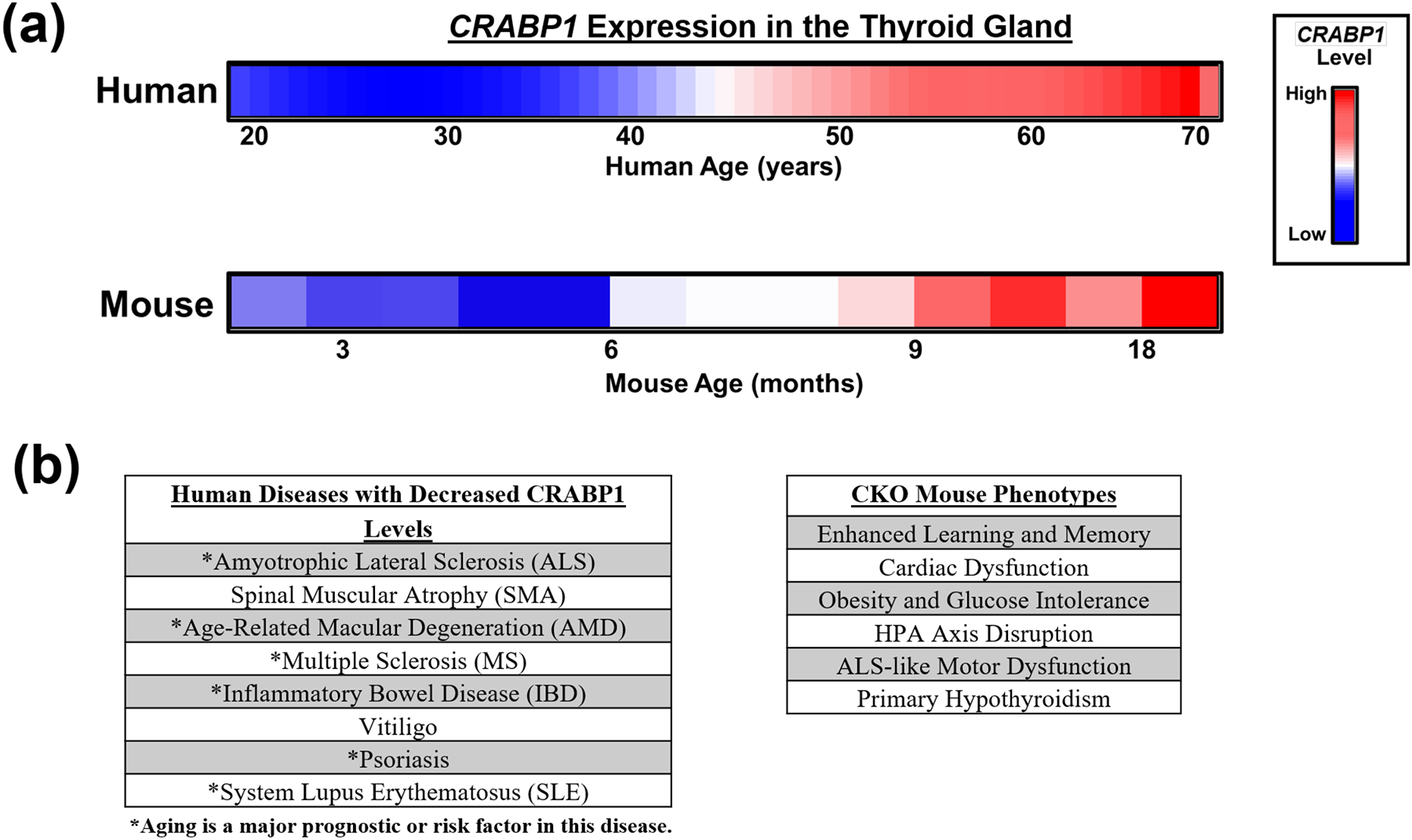
Altered CRABP1 expression in aging and diseases. (**a**) Top: Relative human *CRABP1* gene expression levels in thyroid tissues of healthy people aged 20–70 years old. Human aging data is adapted from the VoyAGER database (https://compbio.imm.medicina.ulisboa.pt/app/voyAGEr, accessed on 14 February 2024) [75]. Bottom: Relative mouse CRABP1 protein levels in normal mouse thyroid tissues aged 3–18 months (unpublished). (**b**) Left: Human diseases where *CRABP1* gene expression is reduced. Asterisks mark diseases where aging is a major prognostic or risk factor. Right: Documented CKO mouse phenotypes.

The Human Protein Atlas expression database (proteinatlas.org [66]) shows that the *CRABP1* gene is highly expressed in several organs, especially in the thyroid gland and the brain. This is consistent with the dramatic thyroid defects [15] and enhanced memory function of CKO mice [10]. Interestingly, CKO mice develop spontaneous hypothyroidism only in mid- to late life (older than 6 months), suggesting the age-dependent expression (or requirement) of this gene in the thyroid gland [15]. Indeed, human *CRABP1* gene expression in the thyroid gland gradually increases with aging until it reaches a plateau in late life (Figure 2a, top). Mouse *Crabp1* gene expression also increases age-dependently in the thyroid gland (Figure 2a, bottom). The similar, temporally specific patterns of CRABP1 expression in both human and mouse thyroid glands further support the critical need for this protein along animals’ lifespans. Thus, it is tempting to speculate that aberrations in CRABP1 levels within individuals or across time may contribute to the likelihood of thyroid dysfunction onset, especially in the context of aging. Additionally, the presence of genetic variants in the *CRABP1* gene might impair its gene regulation; particularly, certain variants might affect the age-associated increase in *CRABP1* expression (Figure 2a, top). This would be clinically informative, such as in assessing thyroid health during aging. To this end, single nucleotide polymorphisms (SNPs) in the human *CRABP1* gene promoter region have already been identified in cancer and ALS patients [7]. However, the functional relevance of these SNPs to *CRABP1* expression levels remains to be experimentally validated. Based on our studies of CKO mice and human bioinformatics findings, we speculate that dysregulation of the *CRABP1* gene may disrupt the homeostasis of certain organ systems, such as the thyroid, due to the failure of cells to engage the potentially protective functions of CRABP1 signalosomes.

Although *CRABP1* expression clearly changes with age in the human thyroid (Figure 2a, top), it remains unclear how intracellular RA levels shift during aging, particularly in human thyroid tissue. Reports have suggested that RA signaling could contribute to thyroid health because vitamin A deficiency could worsen thyroid dysfunction, especially under iodine-limiting conditions [67,68]. Measuring endogenous RA is technically challenging due to its rapid metabolism. Despite these technical challenges, determining the endogenous intracellular RA levels remains an important issue to address.

As to the disease association of CRABP1, it appears that CRABP1 expression changes in many human diseases where age can be an important factor (Figure 2b, left). Particularly, the expression of the *CRABP1* gene is dramatically reduced in ALS and several autoimmune diseases; in both cases, age is a major prognostic or risk factor (Figure 2b) [69–74]. In animal models, CKO mice develop various phenotypes at different stages of the lifespan (Figure 2b, right), further strengthening the notion that CRABP1 can provide certain protective mechanisms in the cells most critically needed for normal function or physiological activities at different stages of the lifespan. While these parallels between human disease and CKO mouse phenotypes are informative, it is important to note that complete gene deletion in mice does not fully reflect the more nuanced or context-dependent changes in *CRABP1* expression seen in these human diseases. Therefore, human diseases involving complex and multifactorial etiologies may not be fully captured by the CKO model. Nevertheless, the worsened phenotypes observed in CKO mice, either spontaneously (e.g., adult-onset hypothyroidism and ALS-like neurodegeneration) or under challenges (e.g., high-fat diet or isoproterenol exposure), would support a broad cytoprotective role for CRABP1 in maintaining tissue function in certain physiological contexts.

## 4. Clinical Relevance of CRABP1 in Thyroid Gland Healthand Dysfunction

Thyroid hormones are essential for the regulation of metabolism in all tissues and organs throughout a human’s lifetime [76]. The production of thyroid hormones in the thyroid gland and their secretion is tightly regulated by the hypothalamus–pituitary–thyroid (HPT) axis, which involves stimulation by thyrotropin-releasing hormone (TRH) that stimulates the anterior pituitary gland to secrete thyroid-stimulating hormone (TSH), thereby inducing synthesis and release of thyroid hormones. The elevated levels of thyroid hormones then suppress the HPT axis, providing negative feedback inhibition to decrease TRH and TSH levels [77,78]. Thyroid dysfunction occurs when the thyroid gland fails to produce appropriate levels of thyroid hormones, leading to disruption of the negative feedback regulation of the HPT axis [79]. The potential roles for CRABP1 in maintaining thyroid health are discussed in the following section.

**Figure 3.**
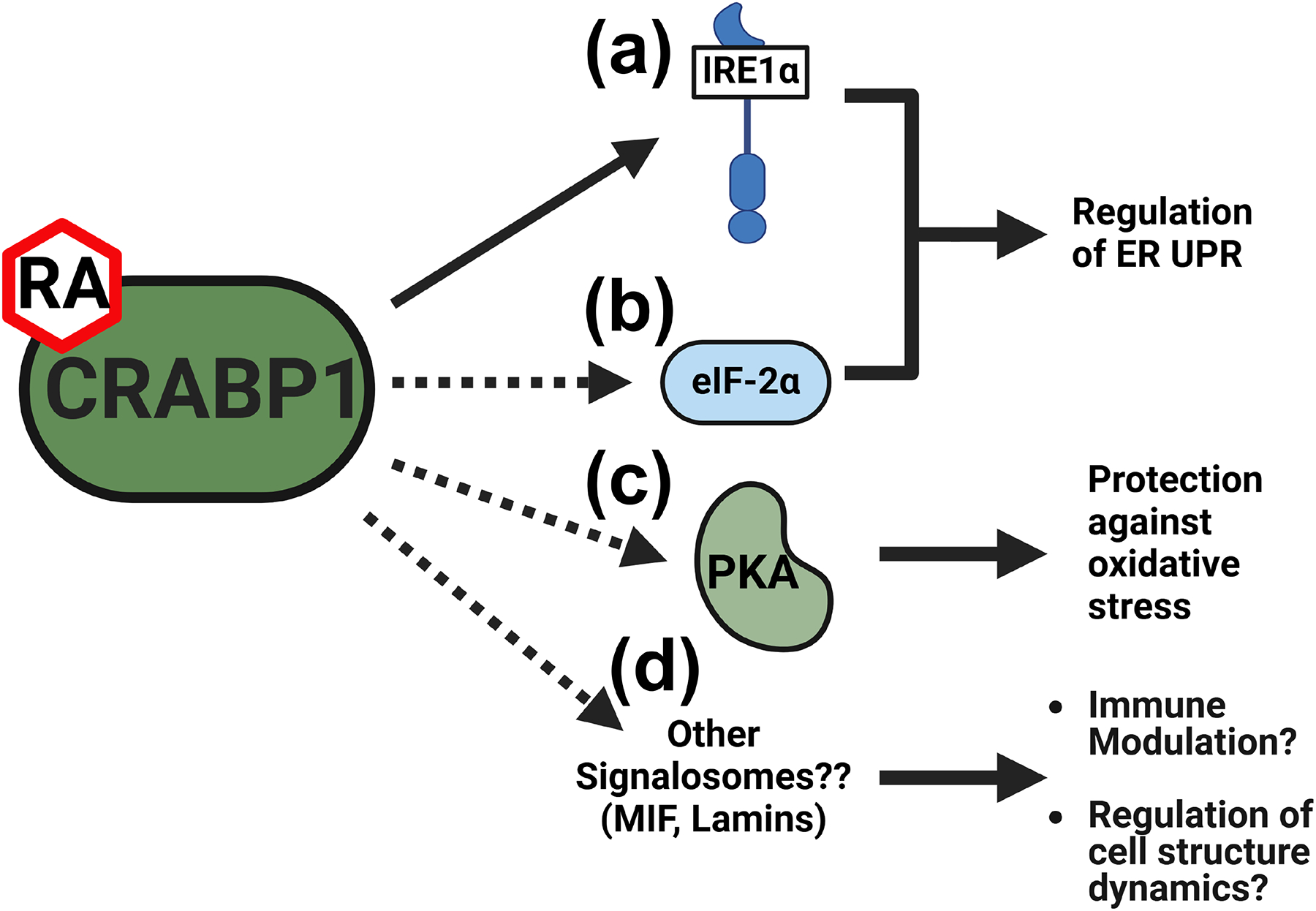
Proposed protective functions of CRABP1 signalosomes in the thyroid gland. (**a**,**b**) CRABP1-IRE1α (**a**) and/or the CRABP1-eIF2α (**b**) signalosome may regulate the ER UPR to protect against ER stress. (**c**) The CRABP1-PKA signalosome may protect against oxidative stress to promote thyrocyte survival. (**d**) Other signalosomes candidates identified in IP-MS studies (detailed in Table 1) may modulate immune response or regulate cell structure dynamics to maintain thyrocyte health. Solid arrows indicate evidence at the experimental level for CRABP1 signalosome functions in thyrocytes. Dashed arrows indicate speculated protective functions of CRABP1 signalosomes.

Hypothyroidism often remains undetected until later stages of disease. The etiologies of hypothyroidism can be heterogeneous. Further, epidemiologic studies have shown that hypothyroidism can occur in both iodine-sufficient and iodine-deficient populations, suggesting other factors contributing to the development of hypothyroidism, such as age, sex, and genetic predisposition [80–84]. Given the increased prevalence of hypothyroidism in elderly patients [85,86], there is a greater need for diagnosis/therapeutics of age-related thyroid dysfunction. Several disease mechanisms have been proposed for hypothyroidism, with ER stress emerging as a key contributor to thyroid dysfunction [87,88]. Our preliminary findings identified a CRABP1-IRE1α complex in the mouse thyroid, as well as hyper-phosphorylation of IRE1α in the thyroid gland of CKO mice (see Section 2.4), suggesting the relevance of CRABP1 in ER stress response. Additionally, the *CRABP1* gene is most highly expressed in the thyroid gland (both human and mouse) [15,32,89] and CRABP1 is considered a normal thyroid marker [90], suggesting a potential role for CRABP1 in the thyroid gland [15]. In one of our studies, we employed IP-MS to identify potential CRABP1 interaction partners, which can be candidate CRABP1 signalosome components [17]. Interestingly, several of these candidates are associated with clinical features of thyroid dysfunction or structural abnormalities in human studies (summarized in Table 1). These additional potential CRABP1 signalosome candidates shed light on new avenues to understand the roles of CRABP1 in maintaining thyroid health, and they could potentially serve as new biomarkers or pharmacological targets for thyroid diseases. To illustrate this possibility, we propose a hypothetic model based upon current knowledge about these potential CRABP1-signalosome candidates (Figure 3). Current animal models for hypothyroidism are designed for either congenital hypothyroidism (mutations/knockouts of essential functional thyroid genes) [91–93] or medication- or surgery-induced hypothyroidism [94–97]. CKO mice retain normal thyroid function at young ages and begin to show signs of hypothyroidism only as they age. Therefore, the CKO mouse model provides a unique opportunity to test this model in aging-related hypothyroidism.

In brief, maintaining a sufficient level of CRABP1 signalosomes in the thyroid gland is important for preserving its health/function. Thus, disrupting CRABP1, or its signalosome components such as IRE1α, can be detrimental to the health of the thyroid. Understanding how these CRABP1 signalosomes form and act to maintain a healthy thyroid gland could provide new insights into novel biomarkers and/or potential therapeutic strategies for hypothyroidism.

## 5. Conclusions and Future Directions

The concept of CRABP1 signalosomes was proposed based on extensive experimental data that showed this protein is involved in multiple signaling pathways, mostly in a cell type-dependent manner. The concept has been further validated in studies of CKO models, both in mice and cultured cells, where CRABP1’s functional roles were determined. Extensive molecular/biochemical/biophysical studies of CRABP1 signalosomes in the MAPK and CaMKII signaling pathways determined the principal mechanisms of action and identified the interaction targets of CRABP1. The spectrum of signaling pathways that can be modulated by CRABP1 is expanding, including those involved in growth, excitation, exosome secretion, mitochondrial stress, ER stress, etc. It would be important to uncover additional CRABP1 signalosomes and their disease associations.

Recent studies have begun to demonstrate how certain RA-mimicking synthetic CRABP1-specific compounds, which bind CRABP1 without activating RARs, can modulate CRABP1’s selectivity to certain signaling pathways [106,107]. This would require highly conserved structural changes (for ligand binding) and interaction surfaces (for molecular interaction) of the CRABP1 molecule, which is consistent with its extreme sequence conservation. The expanding spectrum of CRABP1 signalosomes is consistent with its tightly guarded expression, including regulation in various cell types along an animal’s lifespan, the sensitivity to epigenetic factors, and the association with multiple diseases in which age is a prognostic or risk factor, such as hypothyroidism. As to potential therapeutic applications, since CRABP1-specific (no activation of RARs) compounds can be designed to optimize their selectivity towards certain signaling pathways [106,107], it may be a useful strategy to design or identify more signaling pathway-selective (or biased) CRABP1-specific compounds to modulate certain disease-associated signaling pathways. For the thyroid system, this may be possible by applying CRABP1–IRE1α signalosome-biased ligands to enhance the stress response in the thyroid. On a broader scale, targeting selective CRABP1 signalosomes with novel CRABP1-specific ligands may serve as an attractive therapeutic strategy in future clinical applications by optimizing the therapeutic potential without inducing retinoid toxicity.

## Figures and Tables

**Table 1.** CRABP1 signalosome candidates revealed from IP-MS studies that are also implicated in human studies of thyroid dysfunction and structural abnormalities.

Gene Name	Description	Thyroid Gland Phenotype
***EIF2S3* (eIF2γ)**	GTP-binding component of the eIF2 complex (eIF2α, eIF2β, and eIF2γ) that binds the initiator tRNA required for translation initiation [98].	Patients with MEHMO syndrome due to the *EIF2S3* c.820C > G variant presented with hypothyroidism [99].
***LMNA***	An intermediate filament that forms major structural components of the nuclear lamina [100].	Patients with *LMNA* R482W/Q mutations presented with multi-nodal goiter [101]
***MIF***	A pleiotropic cytokine that regulates innate and adaptive immunity by activating monocytes/ macrophages [102].	MIF expression is increased in thyroid tissues of patients with auto-immune thyroid diseases, GD and HT [103].
***PRKACA***	Catalytic subunit of PKA responsible for kinase activity [104].	Stimulating TSHR auto-antibodies, from GD patients, activate PKA which is associated with increased hormone production, resistance to apoptosis, and prolonged thyrocyte survival [105]. Blocking/neutralizing TSHR auto-antibodies fail to activate PKA and induce oxidative stress-induced thyrocyte apoptosis [105].

**Abbreviations:** “*EIF2S3*”—eukaryotic translation initiation factor 2 subunit 3/gamma; “GTP”—Guanosine-5′-triphosphate; “tRNA”—Transfer ribonucleic acid; “MEHMO”—Mental retardation, Epileptic seizures, Hypogenitalism, Microcephaly, Obesity; “*LMNA*”—Lamin A/C; “*MIF”*—Macrophage Migration Inhibitory Factor; “GD”—Graves’ Disease; “HT”—Hashimoto’s Thyroiditis; “*PRKACA*”—protein kinase cAMP-activated catalytic subunit alpha; “TSHR”—Thyroid-Stimulating Hormone Receptor.

## Data Availability

No new data were created or analyzed in this study. Data sharing is not applicable to this article.
